# P-Coumaric Acid Reverses Depression-Like Behavior and Memory Deficit Via Inhibiting AGE-RAGE-Mediated Neuroinflammation

**DOI:** 10.3390/cells11101594

**Published:** 2022-05-10

**Authors:** Xu-Dong Yu, Dan Zhang, Chu-Li Xiao, Yu Zhou, Xing Li, Le Wang, Zhiming He, James Reilly, Zhi-Yong Xiao, Xinhua Shu

**Affiliations:** 1 Department of Physiology, School of Basic Medical Sciences, Shaoyang University, Shaoyang 422000, China; 3910@hnsyu.edu.cn (X.-D.Y.); zd18873359211@163.com (D.Z.); xcl_neuro@163.com (C.-L.X.); zhouyuneuron@163.com (Y.Z.); lixing3971@hnsyu.edu.cn (X.L.); wangle2021@hnsyu.edu.cn (L.W.); 40003@hnsyu.edu.cn (Z.H.); 2 Hunan Engineering Research Center of Development and Utilization of Traditional Chinese Medicine in Southwest Hunan, Shaoyang University, Shaoyang 422000, China; 3 The First Affiliated Hospital, Hengyang Medical School, University of South China, Hengyang 421001, China; 4 Department of Biological and Biomedical Sciences, Glasgow Caledonian University, Glasgow G4 0BA, UK; j.reilly@gcu.ac.uk

**Keywords:** depression, p-coumaric acid, inflammation, AGE-RAGE signaling pathway, network pharmacology

## Abstract

Depression, a mood disorder, affects one in fifteen adults, has multiple risk factors and is associated with complicated underlying pathological mechanisms. P-coumaric acid (p-CA), a phenolic acid, is widely distributed in vegetables, fruits and mushrooms. P-CA has demonstrated a protective role against oxidative stress and inflammation in various diseases, including cardiovascular disease, diabetes and cancer. In the current study, we investigated the protection of p-CA against depression and memory impairment in a corticosterone (CORT)-induced chronic depressive mouse model. CORT administration resulted in depression-like behaviors and memory impairment. P-CA treatment alleviated CORT-induced depression-related behaviors and memory impairment. Network pharmacology predicted that p-CA had multiple targets and mediated various signaling pathways, of which inflammation-associated targets and signaling pathways are predominant. Western blotting showed CORT-induced activation of the advanced glycation end product (AGE)-receptor of AGE (RAGE) (AGE-RAGE) signaling and increased expression of the proinflammatory cytokines interleukin-1 beta (IL-1β) and tumor necrosis factor-alpha (TNFα) in the hippocampus, while p-CA treatment inactivated AGE-RAGE signaling and decreased the levels of IL-1β and TNFα, suggesting that protection against depression and memory impairment by p-CA is mediated by the inhibition of inflammation, mainly via the AGE-RAGE signaling pathway. Our data suggest that p-CA treatment will benefit patients with depression.

## 1. Introduction

Depression is not only a complex, debilitating, disabling and highly prevalent mental illness, but also a serious public health problem that affects over 350 million people across the world, leading to a high personal and socioeconomic burden. The symptoms of depression span a broad spectrum, including sadness, despair, anhedonia, social withdrawal and weight gain. Additionally, impaired memory has been found in depressed patients [1]. However, to date, about 30% of depressed patients are partially or entirely unresponsive to first-line antidepressants (e.g., fluoxetine and paroxetine), which primarily target monoaminergic systems. Although emerging antidepressants, such as ketamine, exert an antidepressant effect even in patients with treatment-resistant depression, their clinical use is limited by side effects, the potential for addiction and memory deficit. Thus, there is a pressing medical need to develop novel antidepressant medications with ideal therapeutic efficacy and fewer side effects.

P-coumaric acid (p-CA), also named trans-4-Hydroxycinnamic acid, is a phenolic compound widely present in vegetables, fruits and Chinese herbs, including *Curcumae Radix, Cinnamomi Ramulus, Citrus Reticulata, Scutellariae Radix*. In addition to its use as an active ingredient in cosmetics, this phenolic compound demonstrates several pharmacological activities in alleviating hepatic injury [2,3], diabetic nephropathy [4,5], lung inflammation [6], myocardial infarction [7], ovarian toxicity [8], breast cancer [9] and vulvovaginal candidiasis [10]. In addition, in the central nervous system (CNS), p-CA possibly can cross the blood–brain barrier [11] and exert a neuroprotective role [12,13,14]. For example, it can reduce neurotoxicity induced by 5-S-cysteinyl-dopamine or Aβ *in vitro* [12,13] and prevent hippocampal neuronal death induced by cerebral ischemia-reperfusion in mice [14]. Additionally, p-CA also plays a functional role in improving cognitive impairment induced by cerebral ischemia or neuroinflammation [15,16]. Importantly, a possible antidepressant-like role of p-CA was reported in the lipopolysaccharide (LPS)-induced acute depressive model of rats [17]. However, it is still necessary to further evaluate its antidepressant effects and mechanisms in a more effective classic chronic depression model.

Clinical and preclinical studies have shown that the overactive hypothalamic–pituitary–adrenal (HPA) axis and increased serum cortisol levels are common features in depressed patients and depressive animal models [18]. Corticosterone (CORT) is an important mediator in the HPA axis and demonstrates proinflammatory or anti-inflammatory responses depending on circumstances; for example, under chronic stress, CORT displays proinflammatory effects [19]. In addition, chronic corticosterone (CORT) treatment has been reported to induce depressive-like behaviors, including despair, anhedonia and social withdrawal, as well as memory deficits in behavioral tests [20]. Moreover, it also induces neurochemical changes associated with depression, e.g., brain-derived neurotrophic factor (BDNF) and proinflammatory cytokines, which are significantly altered in the serum of depressive patients [20,21]. Furthermore, the administration of antidepressants reversed these depressive-like behaviors and neurochemical changes [20]. Therefore, the chronic CORT-induced depressive animal model is widely accepted and regarded as a classic depressive model for evaluating the therapeutic potential of antidepressants.

In the present study, we first performed behavior tests to examine the effects of p-CA on depression-like behavior and memory deficits induced by chronic corticosterone injections. Then, we performed a network pharmacology analysis to predict the possible targets and pathways of p-CA involved in depression and memory deficits. Additionally, one of the predicted pathways was verified by biochemical methods.

## 2. Material and Methods

### 2.1. Animals

Male Institute of Cancer Research (ICR) mice aged 8 weeks old (Hunan SJA Laboratory Animal Co. Ltd., Hunan, China) were pair-housed in a temperature and light-controlled room, with access to water and food ad libitum. Before the experiments, mice were allowed to habituate to the housing conditions and were handled daily (5 min per mouse) by the experimenter for one week. The experimental procedures were conducted according to the Guidance for the Care and Use of Laboratory Animals, University of South China.

### 2.2. Drug Administration

P-CA and corticosterone (CORT) were obtained from Shanghai Aladdin Biochemical Technology Co. Ltd. (Shanghai, China). Based on the manufacturer’s guidance, p-CA was dissolved in 0.9% saline containing 10% Tween 80 (vehicle 2) and injected intraperitoneally (i.p.) at a dose of 75 mg/kg body weight, while corticosterone was dissolved in 0.9% saline containing 0.1% DMSO and 0.1% Tween 80 (vehicle 1) and injected subcutaneously (s.c.) at a dose of 20 mg/kg body weight.

### 2.3. Experimental Procedure

**Figure 1:**
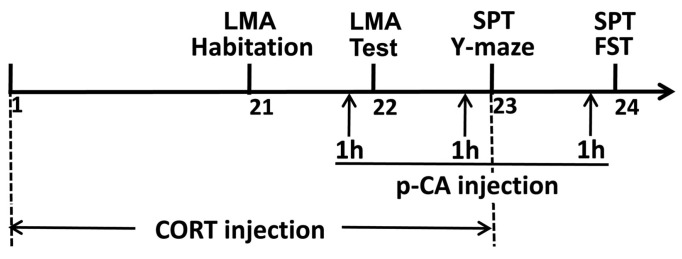
Schematic of the experimental design showing the timeline of drug injection and behavior tasks. CORT, corticosterone; FST, forced swimming test; LMA, locomotor activity; p-CA, p-coumaric acid; SPT, sucrose preference test.

The timeline of behavioral tasks and drug administration is shown in Figure 1. Behavioral tasks were performed in the morning (10:00 to 12:00 A.M.) of day 21 to day 24 (locomotor activity habituation on the morning of day 21; locomotor activity test on the morning of day 22; Y-maze task on the morning of day 23; forced swimming test on the morning of day 24), with the exception of the sucrose preference test that was conducted on the morning and afternoon of days 23 and 24. The depressive model of chronic CORT treatment was established by s.c. injection of corticosterone (20 mg/kg body weight) once a day in the morning for 23 consecutive days, apart from the final 3 days when the injection took place in the afternoon, following the locomotor activity habituation, locomotor activity test or Y-maze, in order to avoid the acute influence of CORT on these behaviors. The CORT-treated mice also received 3 injections of p-CA (75 mg/kg body weight) (CORT + p-CA group, *n* = 10) or vehicle (CORT group, *n* = 10) 1 h before the locomotor activity test, Y-maze or forced swimming test. Meanwhile, mice in the control group were administrated with vehicle 1 (s.c.) for 23 consecutive days without CORT and with vehicle 2 (i.p.) for 3 consecutive days without p-CA for the same period (control group, *n* = 10).

#### 2.3.1. Locomotor Activity (LMA) Task

The LMA task was conducted on the morning of days 21 and 22 (Figure 1). The locomotor activity apparatus consisted of a wooden cabinet (50 × 50 × 60 cm) with attenuated sound (30d) and dim illumination (25Lux) and a video camera mounted on the top of the chamber. Mice were individually placed in the corner of the cabinet and allowed to move freely through the cabinet for a 10-min habituation trial and a 5-min test trial, divided by 24 h. After each trial, the cabinet was cleaned with 75% alcohol in order to eliminate olfactory stimuli. Animal behaviors were videotaped with a digital camera during the 5-min test trial and the locomotor activity was quantified by the total distance traveled, which was analyzed by software Anymaze 6.16.

#### 2.3.2. Y-Maze Task

The Y-maze task was conducted on the morning of day 23. The apparatus of the Y-maze consisted of three horizontal arms (40 cm × 8 cm × 20 cm), positioned at equal angles and labeled A, B, and C. Animals were individually placed at the end of one arm of the Y-maze for 5 min. After each trial, the arms were cleaned with 75% alcohol to eliminate olfactory stimuli. Animal behaviors were videotaped with a digital camera and analyzed by an expert observer under double-blind conditions. Entry into an arm was defined as the mouse placing all four paws on that arm. The number of entries to the arm (A, B or C) was counted. A correct alternation was defined as entry into all three individual arms consecutively (e.g., ABC, BCA or CAB). The locomotor activity was quantified by the total number of arm entries. Spatial memory was quantified by the alternation ratio, which was calculated as (number of correct alternations)/(total number of arm entries − 2).

#### 2.3.3. Forced Swimming Test (FST)

The FST was conducted on the morning of day 24 and the procedure was based on previous studies [16]. Briefly, the FST was carried out in a Plexiglas cylinder (20 cm × 25 cm) located in a sound-attenuating chamber (50 cm × 50 cm × 60 cm) with dim lighting. Mice were individually introduced into the Plexiglas cylinder containing 15 cm-deep water at 25 ± 1 °C for a 6-min test. Animal behaviors were videotaped with a digital camera during the 6-min test and depression-like behaviors of despair were quantified by the immobility time that was scored only for the last 4 min by an observer blinded to the treatment conditions (Figure 1).

#### 2.3.4. Sucrose Preference Test (SPT)

The SPT was conducted over a 48 h period from day 23 to day 24. Two identical bottles containing 1% sucrose solution were presented for 24 h to allow the mice to habituate to them. For the next 24 h, one of the bottles was filled with pure drinking water, while the other remained filled with 1% sucrose solution. All bottles were weighed before and after the 24-h trial to measure the sucrose solution and water consumption. Depression-like behaviors of anhedonia were quantified by the sucrose preference ratio, which was calculated as the formula: sucrose solution consumption/(sucrose solution consumption + water consumption) (Figure 1).

### 2.4. Western Blotting

To avoid the possibility that the behavioral tasks may influence the expression of examined proteins in the current study, another cohort of mice was used with an identical group and procedures as described in Section 2.3, but without participating in any behavioral task. These mice were sacrificed 1 h after the final p-CA injection, and the whole hippocampus tissues were rapidly dissected and frozen at −80 °C. The frozen samples were separately homogenized in radioimmunoprecipitation assay (RIPA) lysis buffer (Applygen Technologies Inc., Beijing, China) containing protease inhibitor cocktail (Merck KGaA, Darmstadt, Germany) for 30 min, then centrifuged at 12,000× *g* for 15 min at 4 °C and the supernatants were collected and kept at −70 °C. Total protein concentration was determined using a bicinchoninic acid (BCA) assay (Beyotime Institute of Biotechnology, Haimen, Jiangsu, China). Subsequently, 50 mg proteins of individual samples were separated on a 10% polyacrylamide gel using electrophoresis and were transferred to polyvinylidene difluoride (PVDF) membranes. The membrane was incubated with primary antibodies targeting advanced glycation endproducts (AGEs), receptors for advanced glycation end products (RAGE), interleukin-1 beta (IL-1β) or tumor necrosis factor-alpha (TNF-α) (Abcam, Cambridge, UK, 1:1000) and β-actin (Proteintech, Rosemont, IL, USA, 1:5000) and secondary antibodies (1:2000). The blots were visualized by the ChemiDoc XRS imaging system (Bio-Rad, Hercules, CA, USA). The amount of each protein was normalized as a ratio of protein/β-actin.

### 2.5. Network Pharmacology Analysis

The potential targets of the p-CA were collected on the Swiss Target Prediction (http://www.swisstargetprediction.ch/, accessed on 30 September 2021) and TargetNet database (http://targetnet.scbdd.com/, accessed on 30 September 2021). Depression and memory impairment-related genes were collected, respectively, by using the Genecards databases (http://www.genecards.org, accessed on 30 September 2021) with keywords “depression” (relevance score ≥ 1) and “memory impairment” (relevance score ≥ 4), respectively. The intersecting targets among p-CA, depression and memory impairment were imported into the STRING database (https://string-db.org/, accessed on 30 September 2021) and Cytoscape 3.7.2 software for protein–protein interaction (PPI) analysis. The intersecting targets were also imported into the R Studio software and the “clusterProfiler” R package to annotate the Gene Ontology (GO) enrichment analysis and Kyoto Encyclopedia of Genes and Genomes (KEGG) pathways. GO enrichment analysis includes a cellular component (CC), molecular function (MF), and biological process (BP), and the top 10 enriched items of GO enrichment analysis were selected. The threshold of *p* < 0.05 was set and the top 20 enriched items of the KEGG pathway were selected.

### 2.6. Statistical Analysis

All data are presented as mean ± SEM (standard error of the mean) and analyzed by Sigma Stat 3.5. Comparisons were made using a one-way analysis of variance (ANOVA), followed by the post hoc comparisons with Tukey’s honestly significant difference test. Results were considered significant if the *p*-value < 0.05.

## 3. Results

### 3.1. P-CA Treatments Alleviate CORT-Induced Depression and Memory Impairments

**Figure 2:**
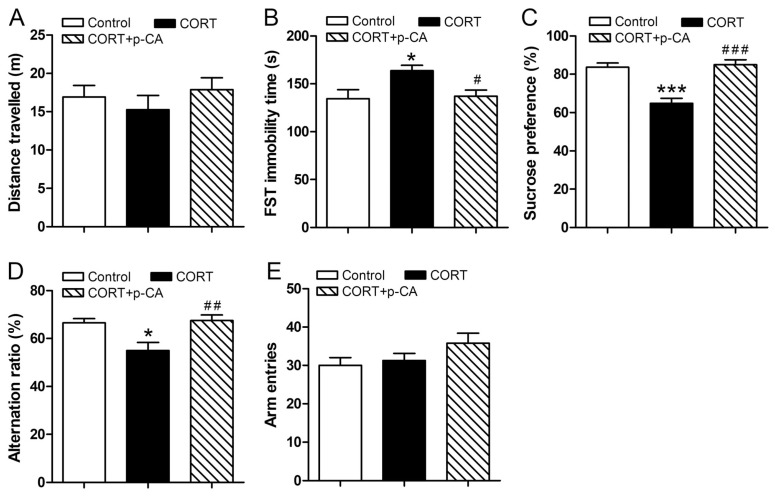
Effect of p-CA treatments on CORT-induced depression-like behaviors and memory impairments. (**A**–**C**) Depression-like behaviors induced by CORT after p-CA treatments. (**A**) Distance traveled in LMA (*n* = 10). (**B**) Immobility time in the FST (*n* = 10). (**C**) Sucrose preference in SPT (*n* = 5). (**D**,**E**) Memory impairments in Y-maze induced by CORT after p-CA treatments. (**D**) Alteration ratio and (E) arm entries in Y-maze (*n* = 10). * *p* < 0.05, *** *p* < 0.001, vs. control group, ^###^ *p* < 0.001, ^##^ *p* < 0.01, ^#^ *p* < 0.05, vs. CORT group. CORT, corticosterone; FST, forced swimming test; LMA, locomotor activity; p-CA, p-coumaric acid; SPT, sucrose preference test.

As a first step in studying the role of p-CA in depressive-like behavior, we investigated the effect of P-CA on CORT-induced chronic depression by utilization of LMA, FST and SPT. In the LMA task, the results showed that CORT-treated animals had reduced travel distance, while co-treatment with p-CA increased travel distance, though there was no significant difference between the groups (*p* = 0.533; Figure 2A). In FST, the results showed that there were significant differences in immobility time among the three groups (*p* = 0.015; Figure 2B). Post hoc comparisons revealed that chronic CORT injection significantly increased the immobility time of mice when compared to the control group (*p* = 0.022), while the p-CA treatment significantly decreased immobility time when compared to that of animals exposed to CORT alone (*p* = 0.039). In SPT, the results showed that there were significant differences in sucrose preference among the three groups (*p* < 0.001; Figure 2B). Post hoc comparisons revealed that chronic CORT injection significantly decreased the sucrose preference of mice when compared to the control group (*p* < 0.001), while p-CA treatment significantly increased the sucrose preference when compared to CORT-injected animals (*p* < 0.001). These results indicate that p-CA alleviated CORT-induced depression-like behaviors.

Next, using a Y-maze task, we investigated the effect of p-CA on depression-related memory impairment in the chronic CORT-induced depressive model. As shown in Figure 2D,E, the results demonstrated that there were significant differences in the alteration ratio (*p* = 0.003; Figure 2D) among the three groups. Post hoc comparisons revealed that chronic CORT injection significantly decreased the alteration ratio of mice when compared to untreated animals (*p* = 0.011), while the alteration ratio was significantly increased in p-CA treated animals when compared to animals exposed to CORT alone (*p* = 0.006). Additionally, chronic CORT injection or co-treatment with p-CA did not influence the arm entries of mice (*p* = 0.167; Figure 2E). These observations suggest that p-CA alleviated chronic CORT-induced memory impairment.

### 3.2. Network Pharmacology Predicts p-CA Targets

**Figure 3:**
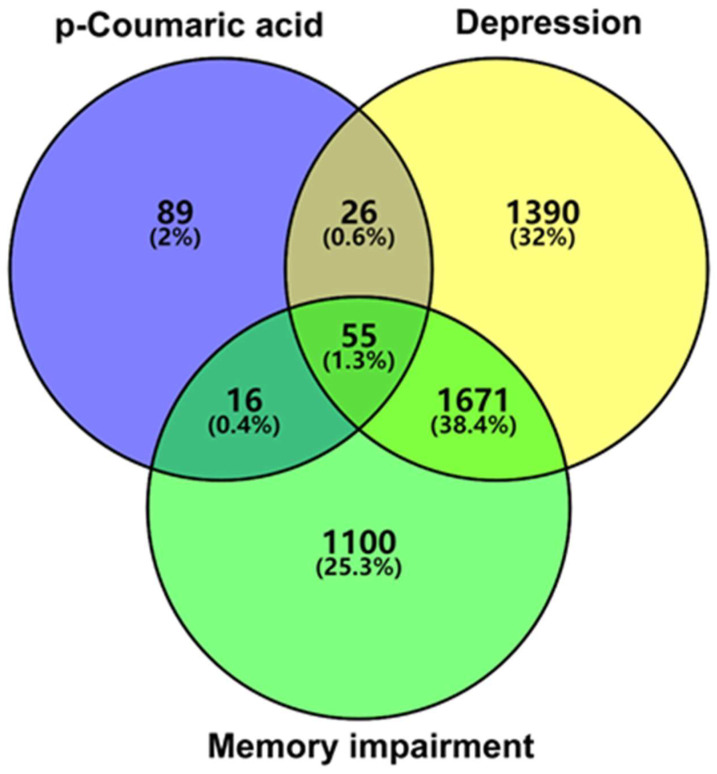
The intersection of p-coumaric acid targets, depression targets and memory impairment targets.

**Figure 4:**
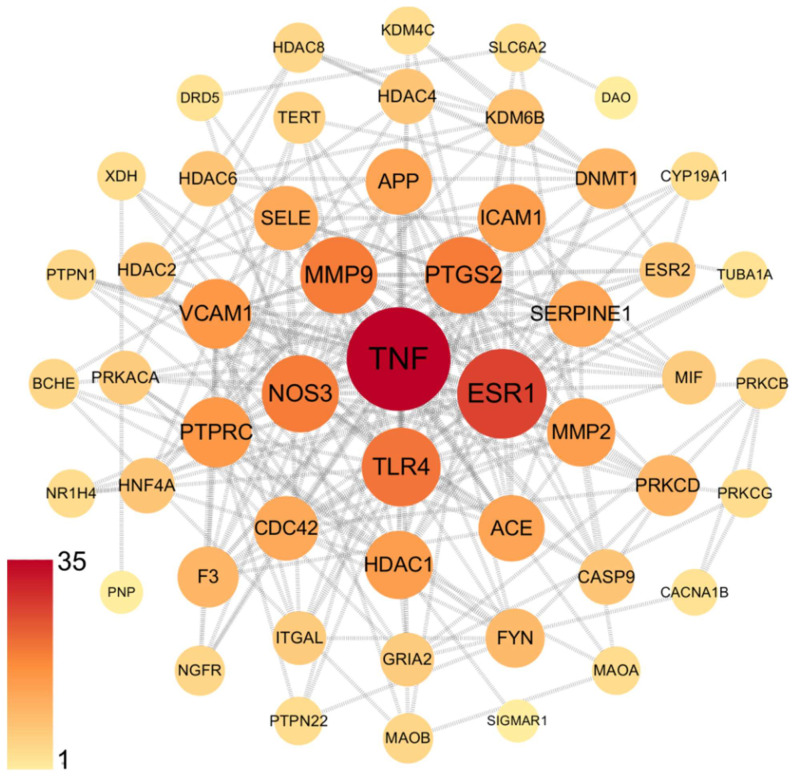
Protein–protein interaction (PPI) network analysis showing 52 intersection targets. The size of the node degree value is indicated by the size and color of the nodes. The larger the node and the deeper the color red represents the higher degree value of the node.

**Figure 5:**
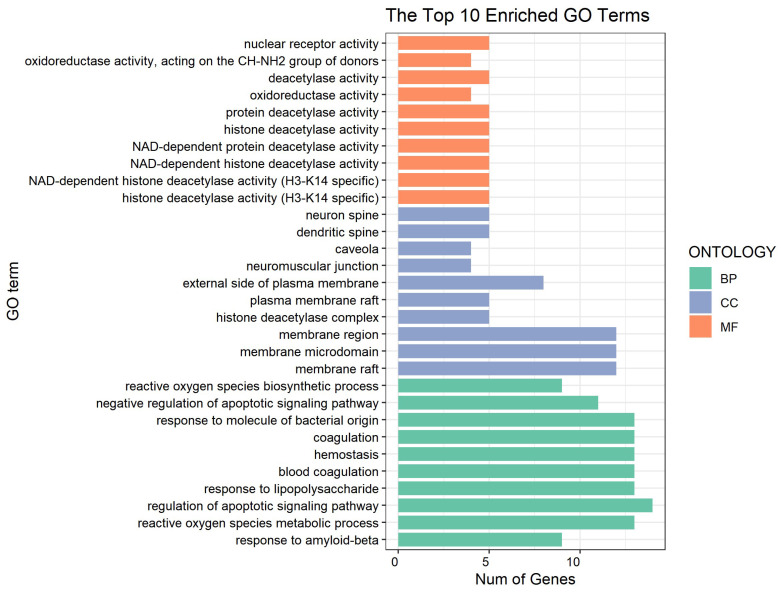
Gene ontology (GO) enrichment showing the top ten enriched terms in three categories: molecular function (MF), cellular component (CC) and biological process (BP). The x-axis represents the number of enriched genes in each term; the y-axis represents enriched terms in each category.

**Figure 6:**
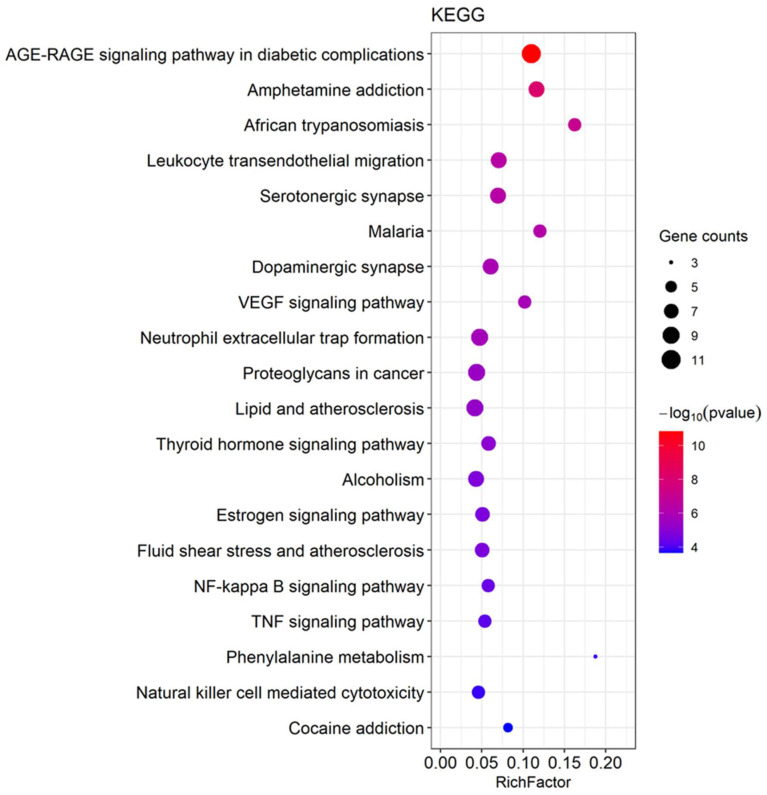
Kyoto Encyclopedia of Genes and Genomes (KEGG) enrichment analysis showing the top 20 of p- coumaric acid (p-CA)-mediated signaling pathways.

To identify p-CA targets and associated molecular mechanisms in depression and memory impairment, we used network pharmacology approaches. A total of 186 genes were identified as the target of p-CA, with duplicate genes removed (Table S1); a total of 3142 genes for depression and 2842 genes for memory impairment were identified, with duplicate genes removed (Table S2). The Venn diagram showed that there were 55 intersecting targets among p-CA, depression and memory impairment (Figure 3, Table S3). Subsequently, a PPI network was constructed using online STRING and Cytoscape tools. Among the 55 common targets, three targets (fat mass and obesity-associated protein (FTO), Retinoic acid-related Orphan Receptor A (RORA) and Folate Hydrolase 1 (FOLH1)) are not shown in Figure 4, because they were not connected to this network. Based on topological analyses, tumor necrosis factor (TNF), estrogen receptor 1 (ESR1), Toll-like receptor 4 (TLR4), prostaglandin-endoperoxide synthase 2 (PTGS2), matrix metallopeptidase 9 (MMP9) and nitric oxide synthase 3 (NOS3) had a degree over 20 and were recognized as crucial nodes in the network (Figure 4). The GO enrichment demonstrated that the targeted genes are mainly involved in nuclear receptor and deacetylase activities that are associated with depression and therapeutic targets for depression treatment [22]. Functions of these targeted genes predominantly take place in membrane domain/raft where receptor-mediated signal transductions may participate in depression formation; these targeted genes play cellular and physiological roles in the generation and metabolism of reactive oxygen species, regulation of apoptosis of these target genes, and response to both LPS and amyloid-beta, which have been shown to contribute to the development of depression and memory impairment (Figure 5) [23,24]. Additionally, the KEGG pathway enrichment was also analyzed to elucidate p-CA- mediated molecular mechanisms. These mapped top twenty signaling pathways are displayed in Figure 6. Among these signaling pathways, the AGE-RAGE, vascular endothelial growth factor (VEGF), nuclear factor kappa-light-chain-enhancer of activated B cells (NF-kB) and TNF signaling pathways are highly related to depression formation and progression [24,25,26]. These results suggest that multiple pathways and multiple targets are involved in the effects of p-CA against depression and memory impairments.

### 3.3. P-CA Treatments Inhibited CORT-Induced Activation of AGE-RAGE Signaling Pathway and Release of Inflammatory Cytokines in Hippocampus

**Figure 7:**
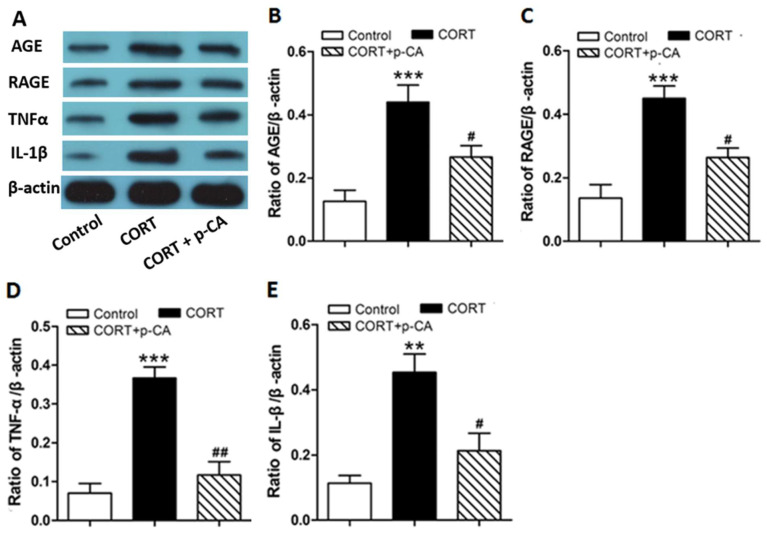
Effect of p-CA treatments on CORT-induced activation of AGE-RAGE signaling and release of inflammatory cytokines in the hippocampus. Representative immunoblots of hippocampal AGE, RAGE, TNFα and IL-1β (**A**) and quantification of these proteins (**B**–**E**) in the hippocampus of control, CORT-treated and CORT + p-CA-treated mice (*n* = 3). *** *p* < 0.001, ** *p* < 0.01, CORT group vs. control group; ^##^ *p* < 0.01, ^#^ *p* < 0.05, CORT + p-CA group vs. CORT group. CORT, corticosterone; p-CA, p-coumaric acid.

We next aimed to further confirm the involvement of the AGE-RAGE pathway in p-CA against depression and memory impairment predicted by network pharmacology analysis. We examined the levels of AGE, RAGE, TNFα and IL-1β by immunoblots. The results demonstrated that the level of these proteins was markedly increased in CORT-treated mouse hippocampus’ when compared to that of control animals; co-treatment with p-CA significantly decreased the levels of these proteins, when compared to animals exposed to CORT alone (Figure 7 and Figure S1). These results indicate that p-CA treatments inhibit CORT-induced activation of AGE-RAGE signaling and the generation of proinflammatory cytokines in the hippocampus.

## 4. Discussion

In the present study, we found that p-CA attenuated chronic CORT-induced despair behavior in FST and anhedonia in SPT, supporting the notion that p-CA has antidepressant effects. P-CA also alleviated the depression-related memory impairment (assessed by Y-maze behavior) induced by chronic CORT injection. Furthermore, a network pharmacology analysis predicted that p-CA mediated multiple targets and signaling pathways, of which the AGE-RAGE signaling pathway was possibly the principal one. Finally, we confirmed activation of the AGE-RAGE signaling pathway and increased release of proinflammatory cytokines in the hippocampus of CORT-treated animals; p-CA treatment counteracted CORT-induced changes.

A recent study used a single LPS injection to induce inflammation and depressive symptoms in rats and demonstrated that p-CA treatment alleviates LPS-induced depression-like behaviors as measured by FST, a tail suspension test (TST) and SPT [17]. Considering that p-CA exerts anti-inflammatory effects, its antidepressant activities are possibly exerted by the inhibition of inflammation. However, a single LPS injection-induced acute depression model is not suitable for the most common depression caused by chronic factors. Our study used the classic chronic CORT-induced depressive model to further confirm the antidepressant-like role of p-CA. Chronic CORT injection elicits depressive-like behaviors, including despair, anhedonia and social withdrawal in rodents [27,28,29] that are comparable with symptoms experienced by depressive patients. Consistently, our results showed that 23-days of CORT injections, while having no significant effect on distance traveled in the LMA test, significantly increased immobility time in the FST and significantly decreased sucrose preference behavior in SPT, indicating that chronic CORT injections resulted in depression-like behaviors and suggesting that this depression model was successfully established in our study. Meanwhile, p-CA significantly attenuated chronic CORT-induced increase in immobility time without influencing the distance traveled in the LMA test, indicating an ‘anti-despair’ role of p-CA; p-CA also significantly attenuated a chronic CORT-induced decrease in sucrose preference, indicating an anti-anhedonia effect of p-CA.

Cognitive dysfunctions are common symptoms in depressive patients and in depressive animal models. Memory impairment has been displayed in chronic stress depressive, acute stress depressive, and drug-induced depressive models [30]. Consistently, our results showed that chronic CORT injection significantly decreased the alteration ratio in the Y-maze test without influencing the arm entries, an index reflecting locomotor activities, suggesting that chronic CORT injection resulted in memory impairment in the Y-maze test, an observation that is noteworthy given that memory impairment is a comorbidity of depression. Increasing evidence suggests that p-CA can modulate this memory impairment. For example, previous studies reported that p-CA improved post-cerebral ischemic spatial memory [15] and alleviated scopolamine-induced memory impairment [31]. Additionally, p-CA mitigates LPS-induced memory impairment in Morris water maze and Y-maze tests [16]. In keeping with these findings, the present study further demonstrated that p-CA significantly attenuated a chronic CORT-induced decrease in the alteration ratio without influencing arm entries, indicating a protective role of p-CA against memory impairment induced by CORT.

Network pharmacology demonstrated that p-CA had multiple targets associated with depression and memory impairment. The six predominant targets (TNFα, ESR1, TLR4, PTGS2, MMP9 and NOS3) have been well documented to associate with depression [24,32,33,34,35]. Among these targets, most mediate inflammation, consistent with early reports of p-CA function [36]. Notably, this is the first time that MMP9 has been predicted as a target for p-CA. MMP9 is expressed in the cerebral cortex, cerebellum and hippocampus at a low level under physiological conditions, while its expression is upregulated under increased neuronal activity/plasticity or pathological conditions [34]. MMP9 polymorphism, C1562T, is associated with depression [37]. Increased levels of MMP9 have been reported in the blood of patients with major depression [38,39] and an MMP9 level is associated with depression severity [40]. MMP9 also plays an important role in hippocampus-dependent learning and memory, and inhibition of MMP9 activity disrupts spatial memory [41,42]. It is highly possible that p-CA regulates MMP9 expression and mediates its function—an observation warranting further investigation.

It has been hypothesized that inflammation plays a central role in the development of depression [43,44,45]. Early reports demonstrated that the levels of proinflammatory cytokines, such as IL-1β, IL-6 and TNFα, were significantly increased in the blood of depressive patients [46], while anti-inflammatory agents displayed antidepressant effects [45,46]. Chronic inflammation also can induce depressive-like phenotypes in rodent models [47]. Continuous administration of corticosterone in rodents has been shown to cause inflammation and depressive behaviors [20]. The protective effects of p-CA against depression and memory impairment are possibly mediated by counteracting CORT-induced inflammation. Among the multiple signaling pathways associated with the p-CA anti-depression function, predicted by the KEGG analysis, inflammation-related signaling pathways, such as TNFα, NF-kB and VEGF signaling pathways, have been well elucidated in depression [24,25]. The predominant signaling pathway from the KEGG enrichment is the AGE-RAGE pathway, which has been involved in various pathological conditions, including cardiovascular disease, diabetes, cancer and neurodegenerative disorders [48]. The AGE-RAGE signaling pathway mediates NF-kB activation and upregulates the expression of proinflammatory cytokines (e.g., IL-1β, IL-6 and TNFα) and growth factors (e.g., VEGF) [49], which contribute to depression development and progression [24]. Here, we demonstrated that the AGE-RAGE signaling pathway was involved in CORT-induced depression and promoted the secretion of proinflammatory cytokines (IL-1β and TNFα). P-CA treatment inactivated the AGE-RAGE pathway and decreased the generation of these cytokines in the hippocampus, possibly by reducing AGE formation. In fact, a recent study showed that depressive-like behavior induced by chronic unpredictable stress was possibly mediated by the activation of RAGE signaling in hippocampal microglia [25].

BDNF, a member of the neurotrophin family, plays a key role in the maintenance of neuronal function and is associated with a wide range of neurodegenerative/neuropsychiatric disorders [21,47]. A significantly low level of BDNF in blood has been linked to depression, while pharmacological treatment of the condition increases serum BDNF levels [21]. Targeting the BDNF-TrkB (tropomyosin receptor kinase B) signaling pathway is a promising strategy to develop novel drugs for the treatment of depression [47]. Previous studies demonstrated that BDNF suppressed inflammation in the hippocampus of type 1 diabetic mice by controlling the RAGE-NF-kB pathway [50]. Recently, it has been reported that p-CA upregulated BDNF expression and promoted neurogenesis in the hippocampus of ischemic rats, resulting in improved spatial learning and memory [15]. Consequently, it would be prudent to investigate whether p-CA enhances BDNF expression and regulates AGE-RAGE-NF-kB signaling pathways in CORT-induced depressive mice.

## 5. Conclusions

P-CA attenuated CORT-induced depressive-like behaviors and memory impairment. The protective effect of p-CA was mediated by multiple targets and signaling pathways, of which the AGE-RAGE was possibly the major signaling pathway. CORT activated the AGE-RAGE signaling and p-CA counteracted the effect. P-CA offers therapeutic potential for patients with depression.

## Data Availability

Not applicable.

## Supplementary Materials

The following are available online at https://www.mdpi.com/article/10.3390/cells11101594/s1, Figure S1: The original blot for Figure 7; Table S1: Targets of p-Coumaric acid; Table S2: Targets of depression and memory impairment; Table S3: Intersecting targets.
