# Supplementary figures and images for: P-Coumaric Acid Reverses Depression-Like Behavior and Memory Deficit Via Inhibiting AGE-RAGE-Mediated Neuroinflammation
Source: Cells. 2022 May 10;11(10):1594. doi: 10.3390/cells11101594 (PMC9139330; doi:10.3390/cells11101594)

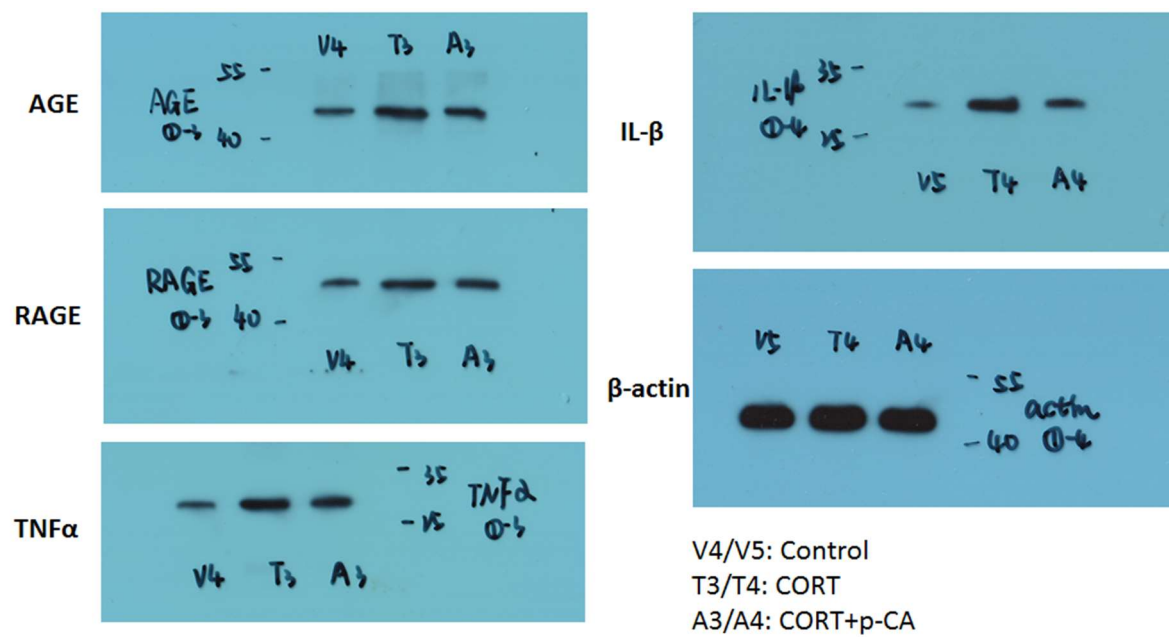

Figure S1: The original blot for Figure 7

Supplement: Supplementary file 1 [file cells-11-01594-s001.zip › cells-1657071 Supplementary conversion/cells-1657071 Supplementary Figure S1.pdf]
